# Supplementary material for: A Bench-Top Approach for Isolation of Single Antibody Producing Chinese Hamster Ovary (CHO) Cells Using a Microwell-Based Microfluidic Device
Source: Micromachines (Basel). 2022 Nov 10;13(11):1939. doi: 10.3390/mi13111939 (PMC9696589; doi:10.3390/mi13111939)
Supplement: Supplementary file 1 [file micromachines-13-01939-s001.zip › micromachines-1959564-supplementary.pdf]

***Micromachines*-1959564 :** A Bench-Top Approach for Isolation of Single Antibody Producing Chinese Hamster Ovary (CHO) Cells Using a Microwell-Based Microfluidic Device

Supplementary material S1

Distribution map of trapped cells in the MBM device using different concentrations of cell suspension (10,000-17,500 cells/mL). 12 tables contain 10 x 20 cells represent the microwells presented in the device. Cells were added into the device from position the right to the left. The cell colored in red represent well with 1 cell. White wells are empty cells.

Cell loading concentration at 10,000 cells/mL

Rep. 1 Total cell trapped number = 418 cells

	1	2	3	4	5	6	7	8	9	10	11	12	13	14	15	16	17	18	19	20
A		2	3		1			1	2	1	2	5	2			3	6	5	4	10
B				2		1	1		1		2		1		2	4	5	3	3	5
C	5			1	4	2	5	1		2	1	6		3	3	5	3	5	6	6
D		3	1	2	1			1	2	3	2	4		1	4	5	2	3	8	11
E	2		3	2	2	1	1	4		3		5		2	3	2		2	6	4
F					1			2	1		2	9		3	2	3	3		6	15
G	1	3	2	1	2	5				1			3	6	2	3	2		5	13
H	2			3	2	2			2	2	2		5			5		3		10
I						1		1	2		1		2	4		6				6
J				2			1		3			2		3	3		3	4	3	7

Rep. 2 Total cell trapped number = 525 cells

	1	2	3	4	5	6	7	8	9	10	11	12	13	14	15	16	17	18	19	20
A		1	2	2	2		2	1	1			4	1	3	3	4			5	9
B			1	4	1	1		2			3	3	4	1	6	2	3	2	4	13
C					5		2		2	5		2		2	4	7		11	12	2
D		5	1	1		2		3		4	1	4		5		8	2	3	10	8
E	2		2		1	6	2	2	1	3		5		6	2	4	3	3	5	15
F			2	2		2	1		4	1		4	1	4	3	4		7		16
G	2	3	1	1	3	9	2	7	5	3	3	3		7	1	5	2	5	4	12
H	6		4	5			1		1			2	2	4	4	9	3	3	3	9
I				1	2	1	2			1	3	4		3	2	7	5	4	2	4
J		1		2			1		3			2		3	3	5		7	3	6

Rep. 3 Total cell trapped number = 486 cells

	**1**	**2**	**3**	**4**	**5**	**6**	**7**	**8**	**9**	**10**	**11**	**12**	**13**	**14**	**15**	**16**	**17**	**18**	**19**	**20**
**A**		1				1	3	1	1		2					6	4	3	7	11
**B**				2	1	1			1		1	2		6	1	5		4	3	9
**C**		3		2		2				3	1		2	5		4	5	4	12	11
**D**		2	1		2		4		2				1		4	7	3	6	7	19
**E**	5	4	3		8			3		1	2	3	2	6	4	5	3	5	4	12
**F**	1			2		1	1			2				4		7	4	8	3	8
**G**	1		1	4	6		1		1			4		3		8	4	9	13	9
**H**	2						2	4					1		5	5		5	6	9
**I**				1	2	3			5	2		1			3	4		7	15	5
**J**						2				1	2	3				6	4	9	5	4

Cell loading concentration at 12,500 cells/mL

Rep.1

Total cell trapped number loading = 701 cells

	**1**	**2**	**3**	**4**	**5**	**6**	**7**	**8**	**9**	**10**	**11**	**12**	**13**	**14**	**15**	**16**	**17**	**18**	**19**	**20**
**A**			3	1	3	1	1	2		1	1	1	2	1	2		1	7	4	18
**B**	1	3	5	3	2	5		1		3	2			4	3	7	4	7		15
**C**	2		3	1	2	1	2	1	1	3	1	2	1	4	4	1	7	8	15	17
**D**	1	4	4	1	3	2	1		3	1	1	3	3		7	8	6	10	8	19
**E**	1	5	1	3	1	5	1	2	1	2	4	2		1	4	9		8	9	10
**F**		3	6	9		1		3	2	1	2	1	3		5	9	7	12	9	9
**G**		4	1	2	1	1	2		1	2	1	3	4		6	6	15	8	8	12
**H**		5	4	3	2	1		1	1	2		2	1	1	5		5	9	17	
**I**	3		3	1	3		2	4	3	1	3	1	1	3	5	8	14	3	7	15
**J**	1	5	2	1		3	1	1		1	4		1	2	3	9	5	5	15	

Rep. 2

Total cell trapped number loading = 855 cells

	**1**	**2**	**3**	**4**	**5**	**6**	**7**	**8**	**9**	**10**	**11**	**12**	**13**	**14**	**15**	**16**	**17**	**18**	**19**	**20**
**A**	1		2	1	4	4	3	1	3	1			5	3	1	4	5	11	19	16
**B**		1		1		1	1	3	1		3	1	5	15	4		9	17	17	14
**C**	4	3	1	3	1	3	1	1	1	1				6	6	8	10	14	16	18
**D**	2	1	1	3		1	3	1	4		1	1		1	8		11	16	15	20
**E**		4	5	2	1	4	1		3	1	1	5	3	2	9	5	8	17	18	7
**F**	1	4	3	5		5		4	1	3	2	3	5	5	5	9	18	11	9	11
**G**	2		1	1		1		1		1	5	1		1	4	10	5	18	8	10
**H**	1	7	3		5	1	3	5	1	1	1	4			5	5	6	19	9	9
**I**		3	5	3	4			1	1	1		1	3	5	3		7	8	17	13
**J**		2	1		1		1	2		3	1	3	3	1	3	8	6	6	10	12

Rep. 3

Total cell trapped number loading = 894 cells

	1	2	3	4	5	6	7	8	9	10	11	12	13	14	15	16	17	18	19	20
**A**	4	1			1		1	3	1		1		6	1	5	14	9	10	9	20
**B**		3	1	1	1		4	1	4		1	5	9	9	6	9		15	9	18
**C**	6	5	3	1		4	1	2		1	1		1	5		13	7	8	10	9
**D**	2	1	1	5	6	1	6	3	3	4	5	1	4	3	9	6	14	6	11	10
**E**		3	2	1	4	5		2	1	4	1	1	5		1	1	16	16	15	15
**F**	3	1		3	1	1	1	1	5	1				1	6	9	17	15	16	14
**G**			1	3	3	6	1	1	1	4	6		1	3		14		13	14	9
**H**		3		2	1	5	6	4		3	1	5	6	3	7	5	15	19	15	15
**I**	2	1	4	1	1	4	7	3		4		1		6	1	3	8	4	19	20
**J**		2			4	1	3	1		1			1	7		1	16	9	11	5

Cell loading concentration at 15,000 cells/mL

Rep. 1

Total cell trapped number = 1,114 cells

	1	2	3	4	5	6	7	8	9	10	11	12	13	14	15	16	17	18	19	20
**A**	1	1	1	1	1		5	1			1	1	3	1	3	16	3	9	12	15
**B**		5	5	9	4	6	2	8	4	1	4	4	5	1	9	14	12	10	24	10
**C**	1	1	1	7	1	3	1	1	1	1		4	1	5	14	13	12	13	14	25
**D**		1	3	1	1	4	4	6	1	1	9	3	15	12	23	13	13	14	14	23
**E**	1	1	1	6	1	3	3	7	1	1	9	1	1	3	12	15	9	13	13	29
**F**		1	2	8	7	3	5	1	4		4	1	1	14	12	13	16	1	4	38
**G**	1	5	1	3	9	6	9	1	1	1	1		1	15	1	4	15	5	12	5
**H**	6	8	3	9	5	5	3			1	3	4	1	4	1	7	3	1	3	10
**I**		6	1	4	1	5	2	3	1		6	7	4	3	3	13	19	5	12	9
**J**		5	1	1	1	1	1	1	1			1	13	9	1	8	14	2	15	5

Rep. 2

Total cell trapped number = 1,017 cells

	1	2	3	4	5	6	7	8	9	10	11	12	13	14	15	16	17	18	19	20
**A**		5	5	1	1	1			2		1	1	2	5	8	17	15	16	16	19
**B**		1	2	1	1	4	1	5	6		1		3	1	3	1	1	10	17	25
**C**	1		3	5	2	2	1	3	2	1	3	3	2	2	1	13	13	13	16	32
**D**	1	1	3	8	8	1	2	1	5	4	1	3	1	4	8	3	13	12	15	30
**E**	1	1	6	3	1		1	3	2	7	3	4	5	9	1	18	14	16	10	16
**F**	2	5	3	2	1	1	1	8	9	2	1	2	1	1	8	9	12	10	13	6
**G**	5	7	8	1	1	3	2	1	2	1	3	6	7	9	4	1	9	9	18	5
**H**	3	1	1	2	1	2	1	2	3	1	3	5	2	1	4	1	15	12	18	9
**I**	1	1	1	2	1	1	8		1	3	1		9	9	5	12	4	13	9	15
**J**		5			1	5	6	2		2			1	5	6	1	3	14	11	12

Rep. 3

Total cell trapped number = 1,073 cells

	**1**	**2**	**3**	**4**	**5**	**6**	**7**	**8**	**9**	**10**	**11**	**12**	**13**	**14**	**15**	**16**	**17**	**18**	**19**	**20**
**A**		6	2	1	1	2	8	2	1		1	1		8	18	7	15	16	15	16
**B**		1	8	1	1	1		1	1	8	1	1	2	12	13	1	1	17	13	15
**C**	1	1	4	9	9	3	2	1		1	4	3		3	1	3	3	1	19	12
**D**	1	1	6	9	1	9	4	1	1	6	1	3	3	1	1	13	13	9	16	30
**E**	1	1	8	2	1	1	1	4		4	2	1	2	1	3	18	14	16	16	16
**F**	2	1	5	1		7	1	9	8	1	7	1	14	5	13	13	12	18	16	19
**G**	7	5	1	1	1		1	3	2		1	4	1	1	1	1	4	23	19	18
**H**	1	1	6	2		1		1	1	2	2	15	1	9	3	1	1	9	15	12
**I**	1	1		1	1	1	1	1	3	3	3	2	14		2	12	14	13	8	17
**J**	1		7	1	9	7		5	1	2		9	8	5		6	13	4	14	14

Cell loading concentration at 17,500 cells/mL

Rep.1

Total cell trapped number = 1,318 cells

	1	2	3	4	5	6	7	8	9	10	11	12	13	14	15	16	17	18	19	20
**A**	5	6	5	5	5	4	4	6	6	8	5	9	7	6		17	5	18	6	16
**B**	6	5	3	7	1	5	6	4	3	9	6	2	8	11		11	4	14	7	15
**C**	5	3	5	1	1	7	1	1	9	4	1	1	8	2	5	4	3	30	16	20
**D**	7	5	9	2	1	6	8	8	8	1	6	1	12	14	5	4	13	24	15	25
**E**	5	1	6	1	1	8	5	5	7	1	1	6	5	1	12	6	4	19	10	26
**F**	8	5	3	2	4	3	5	7	1	6	7	8	11	4		9	2	14	14	17
**G**		3	7	3	7	5	4	7	1	4	3	1	5	8	1	3	14	13	15	15
**H**	8	3	4	5	1	6	5		4	8	4	1	1	11	4	3	5	20	8	14
**I**	7	4	1	1	5	4	3	2	1	5		7	1	2	9	3	14	15	19	19
**J**	4	6	4	3	1	1	4	5	1		8	3	1	4	16	5	13	16	16	20

Rep. 2

Total cell trapped number = 1,386 cells

	1	2	3	4	5	6	7	8	9	10	11	12	13	14	15	16	17	18	19	20
**A**	5	1	5	7	1		1	4	3	6	1	2	7	4	16	7	15	8	4	20
**B**	6	7	6	8	4	5	1		5	8	1	4	9	13	3	11	14	6	17	23
**C**	8	5	3	4	4	4	1	4	1	7	3	3	10	12	1	14	13	20	17	22
**D**	7	4	4	3	1	5	1	6	5	3	5	15	2	4	1	7	13	14	16	25
**E**	4	3	7	4	8	3	9	1	1	3	4		10	4	2	8	14	9	12	36
**F**	7	1	5	1	5	9	1	6	5		2	1	9	1	4	8	23	24	6	27
**G**	6	1	7	4	4	9	6	9	4	5		5	1	1	3	8	16	23	5	35
**H**	7	7	1	1	4	8		1	1	4	1	7	9	4	7	9	4	10	18	4
**I**	8	6	9	5		9	6	1	3	3	3		9	5	19	13	16	5	9	9
**J**	5	9	8		4	4	3		3	2			6	14	6	15	16	6	26	10

Rep. 3

Total cell trapped number = 1,496 cells

	**1**	**2**	**3**	**4**	**5**	**6**	**7**	**8**	**9**	**10**	**11**	**12**	**13**	**14**	**15**	**16**	**17**	**18**	**19**	**20**
**A**	1	7	1	10	3	4	5	1	2	5	5	1	1		6	17	5	2	14	10
**B**	3	2	5	2	1	5	3	4	8	1	10	5	7	3	13	21	12	16	20	34
**C**	7	2	9	5	3	1	6	5	4	5	5	1	15	12	2	12	16	30	7	12
**D**	4	3	1	3	1	6	8	8	1	3	1	8	12	14	3	17	14	24	6	25
**E**	8	7	1	3	5	1	1	1	4	4	3	12	13	5	12	18	4	19	2	25
**F**	2	1	3	6	1	2	4	7	8	2	1	5	5	10	14	6	13	14	30	20
**G**	2	4	9	6	5	9	3	4	1	9	4	6	4	4		19	13	23	15	30
**H**	1	7	5	4	8	1	4	3	3	6	5	1	7	3	17		14	12	21	16
**I**	4	9	8	3	2	4	9	7	5	1	1	6	8		9	5	14	15	23	19
**J**	6	4	1	8	3	5	1	4	4	8	5	5	16	4	16	5	6	8	16	13

Supplementary material S2

Table S1 the raw data of the optimization of single cell trapped (%) using different cell loading concentration (cells mL^{-1}).

Cell loading concentration (cells mL^{-1})	Single cell trapped (%)				
	#1	#2	#3	AVG	STV
**10,000**	12.5	13	12	12.5 [a]	0.5
**12,500**	25.5	25.5	25	25.3 [b]	0.3
**15,000**	31	28	34	31 [c]	3
**17,500**	15.5	14	14	14.5 [a]	0.9

Table S2 the raw data of the optimization of single cell trapped (%) using different settle time (min).

Settle time (min)	Single cell trapped (%)				
	#1	#2	#3	AVG	STV
**1**	11	7.5	12	10.2 [a]	2.4
**3**	16	14.5	17.5	16 [b]	1.5
**5**	32.5	32	32	32.2 [c]	0.3
**7**	19.5	17	16	17.5 [a]	1.8
**9**	4.5	4.5	5	4.7 [d]	0.3

Table S3 the raw data of efficiency of single cell isolation using limiting dilution and the microwell-based microfluidic device.

Techniques	Single cell trapped (%)											
	#1	#2	#3	#4	#5	#6	#7	#8	#9	#10	AVG	STV
**Limiting dilution**	4.5	6	5	6	5	5.5	6	3.5	5	5	5.2	0.8
**The MBM device**	21	28.5	23	25	28.5	17.5	15	29.5	27.5	27.5	24.3***	4.8

Table S4 the raw data of time spent processing using limiting dilution.

Tasks	Time (min)											
	#1	#2	#3	#4	#5	#6	#7	#8	#9	#10	AVG	STV
**Cell preparation**	10.2	5.2	4.5	10.3	5.7	6.1	5.3	4.5	5.8	5.2	6.3	2.2
**Cell loading**	3.2	3.4	5.1	4.2	3.3	3.3	2.5	4.2	2.4	3.2	3.5	0.8
**Single cell assessment**	31.2	15.2	12.3	12.5	12.8	22.3	18.1	31.2	12.3	13.5	18.14	7.6
**Total time-spent processing**	44.6	23.8	21.9	27	21.8	31.7	25.9	39.9	20.5	21.9	27.9	8.3

Table S5 the raw data of time spent processing using microwell-based microfluidic device.

Tasks	Time (min)											
	#1	#2	#3	#4	#5	#6	#7	#8	#9	#10	AVG	STV
Cell preparation	2.4	2.4	2.3	2.4	2.4	2.8	2.37	2.3	2.4	2.3	2.4	0.1
Cell loading	8	8.1	8.2	8.1	8.55	7.3	7.3	7	7	8.1	7.7	0.6
Single cell assessment	14.5	8.2	5.1	8.5	5	12	8.5	5.2	9.2	8.4	8.5	3
Total time-spent processing	24.9	18.7	15.6	19	15.95	22.1	18.17	14.5	18.6	18.8	18.6	3.1

Table S6 the raw data of single cell trapped correctness (%) between experienced and novice testers performing limiting dilution.

Testers	Single cell determination accuracy (%)						
	#1	#2	#3	#4	#5	AVG	STV
Experienced	100	83	90	83	70	85.3	9.8
Novice	85.7	91.8	100	100	100	73.2	16.3

Table S7 the raw data of single cell trapped correctness (%) between experienced and novice testers performing microwell-based microfluidic device.

Testers	Single cell determination accuracy (%)						
	#1	#2	#3	#4	#5	AVG	STV
Experienced	85.7	91.8	100	100	100	95.5	5.8
Novice	100	97.6	97.6	54.6	100	89.9	17.7
